# Efficacy of celery (*Apium graveolens* L.) alcoholic extract against systemic methicillin-resistant *Staphylococcus aureus* infection in rat models

**DOI:** 10.14202/vetworld.2022.898-905

**Published:** 2022-04-12

**Authors:** Yos Adi Prakoso, Agustina Dwi Wijayanti

**Affiliations:** 1 Postdoctoral Program, University of Gadjah Mada, Yogyakarta 55281, Indonesia; 2 Department of Pharmacology, Faculty of Veterinary Medicine, University of Wijaya Kusuma Surabaya 60225, Indonesia; 3 Department of Pharmacology, Faculty of Veterinary Medicine, University of Gadjah Mada, Yogyakarta 55281, Indonesia

**Keywords:** celery, efficacy, healing, methicillin-resistant *Staphylococcus aureus*, systemic infection

## Abstract

**Background and Aim:**

The coronavirus disease-19 (COVID-19) pandemic caused global economic and health problems. The pandemic increased the number of infectious diseases categorized as neglected diseases, such as staphylococcosis, which is caused by methicillin-resistant *Staphylococcus aureus* (MRSA). Celery is an herb that consist of antioxidants that can potentially act as antimicrobial agents. This study aimed to analyze the efficacy of celery alcoholic extract against systemic MRSA infections in rat models.

**Materials and Methods:**

In this study, 36 male, 6-month-old Sprague-Dawley rats (average weight: 300 g) were used as models. The rats were divided into six groups: Group K− (negative control), Group K+ (infected with MRSA without therapy), Group V (infected with MRSA+100 mg vancomycin per kg body weight [BW]), Group P1 (infected with MRSA+1 mg celery extract per kg BW), Group P2 (infected with MRSA+2 mg celery extract per kg BW), and Group P4 (infected with MRSA+4 mg celery extract per kg BW). The therapy was given once daily for 7 days. Blood and organs were taken on day 7 for hematology, serology, immunohistochemistry, and histopathology.

**Results:**

Results showed that 4 mg celery extract per kg BW promotes the healing of MRSA systemic infections in rat models (p≤0.05). The better prognosis was indicated by the normalization of red blood cell indices, white blood cell, neutrophil and lymphocyte counts, Cluster of differentiation 4+, Cluster of differentiation 8+, and Cyclooxygenase-2 expression and absence of severe tissue damage. Celery extracts inhibited MRSA growth in the blood samples.

**Conclusion:**

It can be concluded that celery alcoholic extract can potentially be used as an antimicrobial agent against systemic MRSA infections. A clinical study regarding the efficacy of celery extract must be conducted to ensure its potency against MRSA infections in humans.

## Introduction

The coronavirus disease-19 (COVID-19) pandemic caused global economic and health problems. The high number of daily cases worldwide triggered chaos in health systems [1]. It impacted hospitals to halt health services for diseases with low urgency, such as metabolic diseases and bacterial infections with low pathogenicity. Indirectly, these policies increase the number of infectious diseases, causing them to be categorized as neglected diseases [2]. One of these is staphylococcosis [3].

Staphylococcosis is an infectious disease caused by *Staphylococcus aureus*. Methicillin-resistant *S. aureus* (MRSA) is a common type of staphylococcus that causes severe infections. MRSA infections can occur systemically and trigger massive tissue destruction. 30 cases of MRSA exist with a high fatality rate for every 100,000 people. The major tissue destruction caused by MRSA can lead to meningitis, encephalitis, pneumonia, hepatitis, and systemic inflammation with sepsis [4]. Severe systemic inflammation and sepsis increase the mortality rate among the infected patients. Treatment of MRSA infections becomes harder due to restrictions in massive administration of antibiotics to prevent more complex antibiotic resistance. Nevertheless, vancomycin is the recommended drug for MRSA infections [5]. However, there is worry about generating resistance, sooner or later, due to the widespread use of vancomycin.

The development of herbal-derived therapy must be increased to lessen the use of antibiotics and prevent resistance. Indonesia exhibits high biodiversity, consisting of several species of animals and plants [6]. Several plants contain antioxidants that can potentially be antibacterial agents. One of them is celery (*Apium graveolens* L.). Celery contains terpenoids, flavonoids, alkaloids, saponins, and tannins [7]. These components have been developed as antimicrobial agents due to their ability to inhibit membrane synthesis and destruct bacterial membrane osmolarity [8].

A previous study reported that 1 mg/mL of celery extract promotes membrane damage to MRSA *in vitro* [7]. Further exploration described that celery can be used as a cream-based therapy against MRSA infections in diabetic wounds [9]. However, celery extract has not yet been used against MRSA systemic infections. This study aimed to analyze the efficacy of celery alcoholic extract against MRSA systemic infections in rat models.

## Materials and Methods

### Ethical approval

The animal utilization in this study has been approved by the ethical clearance committee from the Faculty of Dentistry, University of Airlangga, Surabaya, with approval number: 236/HRECC/FODM/V/2020. The committee member conducted monitoring during the study.

### Study period and location

The study was conducted from January 2021 to September 2021. The extraction was performed in the Laboratory of Pharmacology, Faculty of Veterinary Medicine, University of Wijaya Kusuma Surabaya, Indonesia. The determination of herbal biochemical compounds was determined in the Faculty of Health, University of Muhammadiyah Sidoarjo, East Java, Indonesia. The *in vivo* study was conducted in the Laboratory of Pharmacology, Faculty of Veterinary Medicine, University of Gadjah Mada, Yogyakarta, Indonesia.

### Herbal preparation and extraction

Celery was collected from a botanical market in Batu, East Java, Indonesia. The celery species was identified as *A. graveolens* (L.) by a botanist from the Plant Conservation Center, Purwodadi Botanical Garden, East Java, Indonesia. The species was registered with the herbarium voucher no. 0276/IPH.06/HM/II/2019. Drying of the celery was performed using an oven at 80°C for an hour. The dried celery was mashed using a blender and was soaked in 70% alcohol (1:4 dried celery: 70% alcohol). Then, it was evaporated using a rotary evaporator (Buchi R-100, Cat No. 11100v101, Buchi, Indonesia) at 69°C, and the product was stored at 4°C [10].

### MRSA isolate

The MRSA isolate was obtained from the clinical isolates collection of the Laboratory of Bacteriology, Faculty of Health, University of Muhammadiyah Sidoarjo, East Java, Indonesia. Before enrichment, biochemical tests were performed on the isolate. The results of the biochemical tests are as follows: Coagulase (+), DNase (+), hemolysis (+), pigmented colony (+), alkaline phosphatase (+), urease (+), mannitol (+), maltose (+), esculin hydrolysis (−), novobiocin (sensitive), polymyxin B (resistant), and cefoxitin (resistant). The MRSA isolate was enriched using a mannitol salt agar (MSA) plate and was transferred on broth media for incubation until it showed turbidity similar to the 1.0 McFarland standard. The 1.0 McFarland was standardized using a McFarland densitometer.

### Animal model and research design

In this study, 36 male 6-month-old Sprague-Dawley rats (weight: 303.58±10.63 g) were used as models. The rats were obtained from the Laboratory of Pharmacology, Faculty of Veterinary Medicine, University of Wijaya Kusuma Surabaya. Before the treatment, the rats were acclimated to the laboratory for 7 days using several conditions, including, 12/12 h of light/dark, 25°C room temperature, feed, water *ad libitum*, and they were maintained individually. The rats were divided into six groups with six rats each: Group K− (negative control/uninfected and untreated), Group K+ (infected with MRSA without therapy), Group V (infected with MRSA+100 mg vancomycin [Vanconex Iyo, Cat No. J01XA01, Amarox Pharma Global, Indonesia] per kg body weight [BW]) [11], Group P1 (infected with MRSA+1 mg celery extract per kg BW), Group P2 (infected with MRSA+2 mg celery extract per kg BW), and Group P4 (infected with MRSA+4 mg celery extract per kg BW). The celery extract doses were selected following a previous study [7]. MRSA infection was induced by intraperitoneal injection of 50 mL of 1.0 McFarland standard bacterial suspension. The therapy was given 24 h after the injection. Before the therapy, the celery extract was measured and was dissolved in distilled water. The therapy was given once daily for 7 days using a gastric probe.

### Routine hematological test and serology test

After 7 days of treatment, blood and serum were collected from the tail veins of the rats. The blood was tested for red blood cells (RBC), white blood cells (WBC), hemoglobin (Hb), packed cell volume (PCV), mean corpuscular volume (MCV), mean corpuscular Hb (MCH), MCH concentration (MCHC), differential leukocytes, and platelets. The hematology test was performed using an automated hematology analyzer (Medonic M-32 Series, Cat No. CDS1400075, Boule, Sweden). The serum was tested against C-reactive protein (CRP) following a previous study [12].

### Bacteriological examination of blood

MRSA was isolated from the blood of the infected rats. The collected blood was cultured on an MSA plate by directly streaking the blood sample on the MSA surface. The plate was incubated at 37°C for 24 h [13]. The presence of bacterial colonies was considered positive (+).

### Histopathology

After blood collection, the rats were euthanized using lethal doses of dissociative anesthetic (150 mg/kg BW ketamine+10 mg/kg BW xylazine). Necropsy was performed to collect several organs, including the brain, lung, heart, liver, spleen, and kidney. The organs were soaked in 10% neutral buffer formalin (NBF) for 24 h. The tissue was cleared and was dehydrated using xylene and graded alcohol, respectively. The organs were embedded in liquid paraffin and were blocked using a base mold. The blocked organs were cut using a microtome and were placed onto glass slides. The tissue sections were stained using hematoxylin and eosin.

### Cell tube block test and immunohistochemistry (IHC)

The cell tube block test was performed by inserting blood samples into plain capillary tubes. Then, it was centrifuged, and the buffy coat was taken and stored in 10% NBF [14]. Next, the collected buffy coat was processed similar to routine histopathology procedures and was attached to glass slides. IHC staining against cluster of differentiation 4+ (CD4+), cluster of differentiation 8+ (CD8+), and cyclooxygenase-2 (COX-2) monoclonal antibodies was performed following a previous study [9].

### Morphometry

Morphometry was performed by a senior pathologist from the Department of Pathology, Faculty of Veterinary Medicine, University of Gadjah Mada, Yogyakarta, India. The observed lesions were scored as follows: 1=no histopathological changes, 2=mild, 3=moderate, and 4=severe histopathological changes. The pathologist was unaware of the sample treatment and conducted an objective analysis of the samples. The percentage of the cells that expressed CD4+, CD8+, and COX-2 was measured using ImageJ software (NIH, USA, Public Domain, BSD-2).

### Statistical analysis

Several types of data were collected in this study, including quantitative and categorical data. Quantitative data were collected from the hematology profile, leukocyte profile, CRP, and cell tube block and were analyzed using one-way analysis of variance and *post hoc* test. Categorical data from histopathology were analyzed using Kruskal-Wallis and Mann-Whitney U-test. p≤0.05 was considered significant. All statistical analyses were performed using the statistical package for the social sciences v.16 (IBM Corp., NY, USA).

## Results

### RBC indices

Hematology analysis revealed significant differences between the RBC, Hb, PCV, MCV, MCH, and MCHC profiles of the rats systemically infected by MRSA 7 days after therapy (p≤0.05). The routine blood test results showed that treatment with vancomycin demonstrates similar results to treatment with 1 mg and 2 mg celery extract per kg BW in terms of Hb, PCV, MCH, and MCHC. Treatment with 4 mg celery extract per kg BW generated similar results to the negative control group in terms of MCH and MCHC (Table-1).

**Table 1:** RBC indices and platelets count of rats systemically infected by MRSA after the treatment on day 7.

Parameters	Group (mean±standard of deviation)					

	K−	K+	V	P1	P2	P4
RBC (10^6^ cells/µL)	5.61±0.04^a^	4.92±0.20^b^	5.34±0.09^c^	5.37±0.07^c^	5.38±0.07^c^	5.41±0.10^c^
Hb (g/dL)	14.53±0.42^a^	10.60±0.56^b^	11.00±0.59^b^	11.03±0.59^b^	11.58±0.63^b^	13.12±1.13^c^
PCV (%)	41.40±0.77^a^	33.63±1.87^b^	37.55±0.84^c^	37.48±0.59^c^	37.88±0.72^c^	38.88±0.67^d^
MCV (fL)	73.73±1.18^a^	68.31±4.36^b^	70.31±1.47^c^	69.82±1.89^c^	70.31±0.93^c^	71.83±2.61^c^
MCH (Pg)	25.88±0.70^a^	21.52±1.07^b^	20.60±1.16^c^	20.53±0.91^c^	21.50±1.20^c^	24.25±2.69^a^
MCHC (%)	35.10±0.97^a^	31.56±1.75^b^	29.33±2.17^b^	29.44±1.91^b^	30.59±1.86^b^	33.74±3.26^a^
Platelets (×10^5^/µL)	4.38±0.07^a^	2.98±0.35^b^	4.07±0.11^c^	3.93±0.08^c^	4.13±0.11^c^	4.22±0.10^c^

K−=Negative control, K+=Positive control, V=100 mg vancomycin per kg BW, P1=1 mg celery extract per kg BW, P2=2 mg celery extract per kg BW, P4=4 mg celery extract per kg BW, RBC=Red blood cells, Hb=Hemoglobin, PCV=Packed cell volume, MCV=Mean corpuscular volume, MCH=Mean corpuscular hemoglobin, MCHC=Mean corpuscular hemoglobin concentration, MRSA=Methicillin-resistant *Staphylococcus aureus,* BW=Body weight. ^a,b,c,d^ Different superscripts on the same row indicated a significant difference (p≤0.05)

Treatment with vancomycin and all doses of celery extract generated similar results in terms of circulatory platelet count (p≥0.05) (Table-1). Based on this study, vancomycin and all doses of celery extract increased the number of RBC, Hb, and PCV, which indicates a better prognosis during the treatment after being infected by MRSA. However, microcytic hypochromic anemia was still observed in the groups treated with vancomycin and 1 mg and 2 mg celery extract per kg BW. The group treated with 4 mg celery extract per kg BW showed signs of microcytic normochromic anemia (Table-1).

### WBC count

WBC, neutrophil, and lymphocyte count increased more in the in-group positive control compared to the others (p≤0.05). An increase in WBC, neutrophil, and lymphocyte count was also observed in the group treated with vancomycin and celery extract compared to the negative control. Normalization of the lymphocytes/neutrophils (L/N) ratio was found in all the groups treated with celery extract, regardless of the dose, compared to the negative control (p≥0.05) (Table-2).

**Table 2:** WBC and differential leukocytes count of rats systemically infected by MRSA after the treatment on day 7.

Parameters	Group (mean±standard of deviation)					

	K−	K+	V	P1	P2	P4
WBC (×10^3^ cells/µL)	6.39±0.07^a^	9.28±0.49^b^	7.29±0.41^c^	7.41±0.30^c^	7.14±0.16^c^	7.01±0.19^c^
Neutrophil (×10^3^ cells/µL)	1.12±0.11^a^	2.28±0.14^b^	1.83±0.17^c^	1.53±0.16^d^	1.31±0.21^a^	1.16±0.12^a^
Lymphocyte (×10^3^ cells/µL)	4.58±0.09^a^	6.27±0.44^b^	4.80±0.25^a^	5.52±0.32^c^	5.53±0.21^c^	5.39±0.44^c^
Monocyte (×10^3^ cells/µL)	0.62±0.07^a^	0.59±0.23^a^	0.57±0.09^a^	0.24±0.11^b^	0.20±0.08^b^	0.26±0.17^b^
Ratio L/N	4.12±0.43^a^	2.76±0.31^b^	2.62±0.21^b^	3.63±0.41^a^	4.32±0.74^a^	4.65±0.60^a^

K−=Negative control, K+=Positive control, V=100 mg vancomycin per kg BW, P1=1 mg celery extract per kg BW, P2=2 mg celery extract per kg BW, P4=4 mg celery extract per kg BW, L/N=Lymphocyte/neutrophil, WBC: White blood cells, MRSA=Methicillin-resistant *Staphylococcus aureus,* BW=Body weight. ^a,b,c,d^ Different superscripts on the same row indicated a significant difference (p≤0.05)

### CRP level

**Figure-1:**
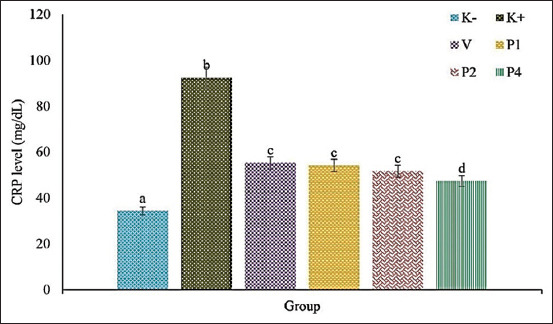
The CRP level of rats infected systemically with MRSA. K−=Negative control, K+=Positive control, V=100 mg vancomycin per kg BW, P1=1 mg celery extract per kg BW, P2=2 mg celery extract per kg BW, P4=4 mg celery extract per kg BW. CRP=C-reactive protein, MRSA=Methicillin-resistant *Staphylococcus aureus*, BW=Body weight.

The CRP levels in the positive control group increased significantly compared to the other groups (p≤0.05). This indicated severe systemic inflammation following artificial MRSA infection in the rat models. CRP levels decreased in the group treated with 100 mg vancomycin per kg BW and with 1 mg, 2 mg, and 4 mg celery extract per kg BW. The group treated with 4 mg celery extract per kg BW exhibited the lowest CRP level, close to the negative control group (Figure-1).

### Bacterial examination from blood

Based on the bacterial examination of the blood, MRSA was isolated from all the blood samples in the positive control group. MRSA was not isolated from the group treated with 100 mg vancomycin per kg BW and 4 mg celery extract per kg BW. MRSA was still isolated from the groups treated with 1 mg and 2 mg celery extract per kg BW with varying results (Table-3).

**Table 3:** Isolation of MRSA from blood of rats systemically infected by MRSA after the treatment on day 7.

Parameters	Group (% positive to MSA)					

	K−	K+	V	P1	P2	P4
MRSA	0/6 (0%)	6/6 (0%)	0/6 (0%)	3/6 (50%)	3/6 (50%)	0/6 (0%)

K−=Negative control, K+=Positive control, V=100 mg vancomycin per kg BW, P1=1 mg celery extract per kg BW, P2=2 mg celery extract per kg BW, P4=4 mg celery extract per kg BW, BW=Body weight, MRSA=Methicillin-resistant *Staphylococcus aureus*

### Histopathology

Histopathology examination of the samples showed various results. The positive control group showed the least histopathological changes (p≤0.05). The most severe histopathological changes were observed in the vancomycin group and in the 1 mg, 2 mg, and 4 mg celery extract per kg BW (p≤0.05) (Table-4) groups. The observed histopathological changes on all the soft tissue were inflammation, necrosis, and hemorrhage. All the histopathological changes observed are summarized in Table-5.

**Table 4:** Histopathology score from the organs of rats systemically infected by MRSA after the treatment on day 7.

Organ	Group (mean±standard of deviation)					

	K−	K+	V	P1	P2	P4
Brain	1.00±0^a^	3.83±0.40^b^	3.33±0.81^c^	2.83±0.40^c^	2.16±0.75^d^	1.83±0.75^d^
Lung	1.33±0.51^a^	4.00±0^b^	3.16±0.75^c^	3.16±040^c^	2.33±0.81^d^	2.16±0.40^d^
Heart	1.16±0.40^a^	3.83±0.63^b^	3.00±0.63^c^	2.83±0.40^c^	2.66±0.51^c^	2.50±0.54^c^
Liver	1.00±0^a^	3.33±0.51^b^	2.66±0.51^c^	2.50±0.54^c^	2.16±0.40^c^	2.00±0.63^d^
Spleen	1.00±0^a^	4.00±0^b^	3.66±0.51^c^	3.00±0.63^d^	2.50±0.54^e^	2.00±0^e^
Kidney	1.16±0.40^a^	3.66±0.51^b^	3.00±0.63^c^	3.00±0.63^c^	2.66±0.51^d^	2.33±0.51^d^

K−=negative control, K+=positive control, V=100 mg vancomycin per kg BW, P1=1 mg celery extract per kg BW, P2=2 mg celery extract per kg BW, P4=4 mg celery extract per kg BW, BW=Body weight, MRSA=Methicillin-resistant *Staphylococcus aureus*. ^a,b,c,d^ Different superscripts on the same row indicated a significant difference (p≤0.05).

**Table 5:** Histopathology from the organ infected systemically with MRSA.

Organ	Histopathology	Figure
Brain	Perivascular cuffing, microgliosis, hemorrhage, neuronal necrosis	2a-d
Lung	Bronchial and interstitial inflammation, hemorrhage, necrosis	3a
Heart	Inflammation, myocardial necrosis	3b
Liver	Inflammation in the intermediate and periportal zone, fatty degeneration, congestion	3c and d
Spleen	Inflammation, necrosis, hemorrhage	3e
Kidney	Tubular necrosis, inflammation, hemorrhage	3f

MRSA=Methicillin-resistant *Staphylococcus aureus*

**Figure-2:**
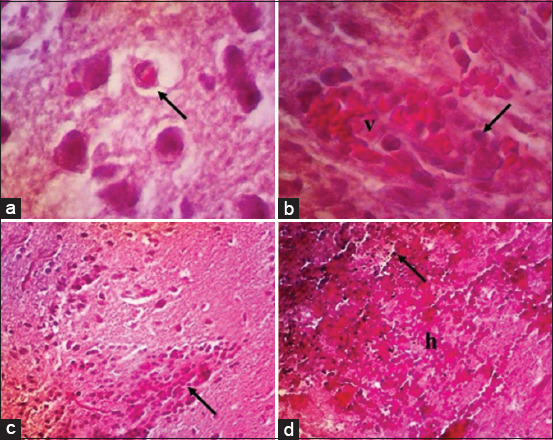
Histopathology of brains from rats infected systemically with MRSA. Neuronal necrosis (arrow) is marked by the reddish cytoplasm within the cerebellum (a); lymphocytes (arrow) surrounding the brain blood vessel (v) within the cerebrum (b); there is an increase of subpopulation microglial cells within the cerebellum (c); and severe extravasation of RBC (h) with bacterial clump (arrow) in the cerebrum (d). Hematoxylin and eosin, 400× (a and b), 100× (c and d). MRSA=Methicillin-resistant *Staphylococcus aureus*, RBC=Red blood cells.

One of the eminent organs impacted by MRSA systemic infection was the brain. Brains from all the infected groups, whether treated and untreated, suffered from several histopathological changes, such as mild to severe neuronal necrosis. Neuronal necrosis occurred significantly in the positive control (Figure-2a). Lymphocytic perivascular cuffing (Figure-2b) and microgliosis (Figure-2c) were observed in all the infected groups. Severe hemorrhage was only observed in the positive control (Figure-2d). Brain histopathology significantly improved along with vancomycin therapy and celery extract dose.

**Figure-3:**
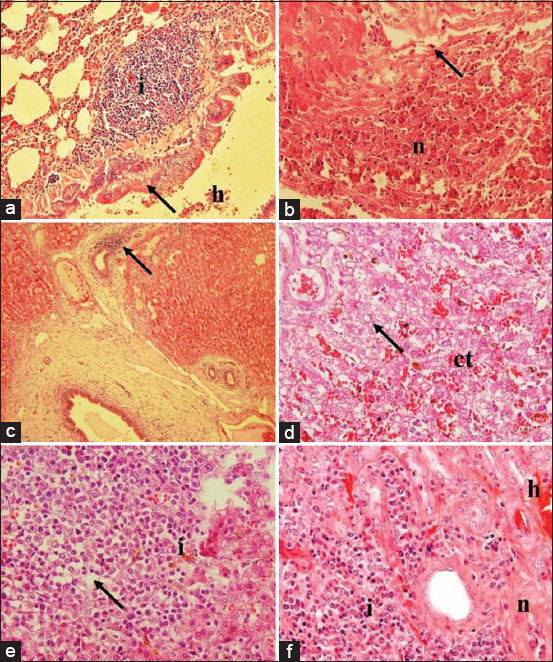
Histopathology of lung, heart, liver, spleen, and kidney from rats infected systemically with MRSA. Predominant infiltration of lymphocyte (i) in the submucosa of bronchus, with hemorrhage (h) and epithelial necrosis (arrow) within lung tissue (a); infiltration of neutrophil (arrow) with severe necrosis of myocardial (n) tissue (b); infiltration of lymphocytes surrounded bile duct (arrow) (c); and dilatation of sinusoid with congestion (ct) and vacuolization of fat within the hepatocytes (arrow) (d); predominant infiltration of neutrophil (i) within the white pulp and it causes either, necrosis and depletion (arrow) of the pulp (e); severe infiltration of neutrophil in the tubulointerstitial area (i) with the tubular necrosis (n) and hemorrhage (h) within the kidney (f). Hematoxylin and eosin, 40× (a and c), 100× (b and d-f). MRSA=Methicillin-resistant *Staphylococcus aureus*.

Besides cerebral lesions, other lesions were also observed in several organs, including hemorrhage bronchopneumonia in the lung (score: 2-4), inflammation and necrosis in the heart (score: 2-4), inflammation in the liver (score: 2-3), inflammation and necrosis in the spleen (score: 2-4), and inflammation and hemorrhage in the kidney (score: 2-3). Massive inflammation, necrosis, and hemorrhage were observed in the lung tissue (Figure-3a). Myocardial necrosis and inflammation occurred in the heart, which indicated systemic infection in the bloodstream (Figure-3b). The liver showed cholangitis, an indication of periportal inflammation (Figure-3c). The intermediate zone was also impacted with congestion and fatty degeneration (Figure-3d). Severe inflammation, pulp depletion, and fatty degeneration occurred in the spleen (Figure-3e) along with severe nephritis (Figure-3f). The observed lesions decreased concomitantly in the group treated with vancomycin and 1 mg, 2 mg, and 4 mg celery extract per kg BW. The decrease in the lesions of the treated groups indicated a better prognosis or mechanism of protection and healing of the visceral organs from bacterial invasion and colonization.

### Expression of CD4+, CD8+, and COX-2

CD4+ is a lymphocyte commonly used as a healing indicator, while CD8+ is a marker used to indicate the immune system’s activity against infections. The treatment either using vancomycin or celery extract beneficially affects on the expression of CD4+ and COX-2. The positive effects of the treatment were indicated by a similar range of percentage of CD4+ and COX-2 between treated and negative control groups (p≥0.05) (Table-6). Furthermore, the group treated with 4 mg celery extract per kg BW showed the best result on the expression of those markers. The significant increase in CD8+ and COX-2 expression in the positive control was aggravated by the CD4+ depletion (p≤0.05) (Table-6).

**Table 6:** Percentage of circulatory CD4+, CD8+, and COX-2 of rats systemically infected by MRSA after the treatment on day 7.

Parameters	Group (mean±standard of deviation)					

	K−	K+	V	P1	P2	P4
CD4+ (%)	3.12±0.13^a^	2.26±0.77^b^	3.16±0.32^a^	2.92±0.18^a^	3.10±0.19^a^	3.53±0.29^a^
CD8+ (%)	2.05±0.26^a^	6.82±0.85^b^	2.96±0.12^c^	3.13±0.22^c^	2.49±0.22^c^	2.22±0.27^a^
COX-2 (%)	2.63±0.29^a^	8.35±0.32^b^	2.66±0.25^a^	2.45±0.32^a^	2.87±0.34^a^	2.48±0.31^a^

K−=Negative control, K+=Positive control, V=100 mg vancomycin per kg BW, P1=1 mg celery extract per kg BW, P2=2 mg celery extract per kg BW, P4=4 mg celery extract per kg BW, BW=Body weight, CD4+=Cluster of differentiation 4+, CD8+=Cluster of differentiation 8+, COX-2=Cyclooxygenase-2, MRSA=Methicillin-resistant *Staphylococcus aureus*. ^a,b,c,d^ Different superscripts on the same row indicated a significant difference (p≤0.05)

## Discussion

MRSA infections can spread systemically and cause severe organ damage. The ability of MRSA to spread in the bloodstream is due to its virulence factors [15]. MRSA exhibits several virulence factors, such as adhesive matrix molecules, biofilm, leukocidin, enzymes, and toxins [16]. During the first infection, MRSA gradually forms a biofilm on host epithelial and endothelial cells. A biofilm is a cluster of bacterial cells surrounded by exopolysaccharides. The formation of a biofilm on the endothelial surface enables MRSA to survive eradication of the immune system [17].

Many biofilms within the circulatory system further allow MRSA to release small colony variants (SCVs). SCVs support MRSA in spreading without causing any severe tissue damage and without overwhelming the immune response. In more advanced cases, SCVs spread in various tissues. As a small colony, their defense mechanism includes producing antiphagocytic microcapsules that allow them to hide from the immune response and from adhesive matrix molecules to prevent opsonization [18]. After they survive inside the host body, MRSA synthesizes adherence proteins [19] and leukocidin concomitantly to interfere with neutrophil movement and make pores on the leukocyte membrane during the infection [20]. High colonization of MRSA releases several types of enzymes, including lipases and proteases, that massively destroy host tissues. Massive tissue destruction triggers severe hemorrhage, activates coagulation, and is aggravated by toxins released by MRSA [21].

The pathogenesis mechanism of MRSA infection can be elucidated from the results of this study. The positive control suffered from microcytic hypochromic anemia, thrombocytopenia, leukocytosis, a decrease in L/N ratio, increased CRP level, severe histopathological changes, and decreased CD4+ expression, and an increase in CD8+ and COX-2 expression. These results agree with the results of a previous study conducted by Prakoso *et al*. [22]. Microcytic hypochromic anemia was the main impact of severe hemorrhage after MRSA infection in the rat models. Severe hemorrhage in the positive control was observed from the histopathology of several collected specimens, such as the brain, lung, liver, and kidney. Following hemorrhage, platelets were activated, and they infiltrated the tissue to minimize RBC extravasation and cause thrombocytopenia [23]. Circulatory MRSA within the bloodstream also acts as a chemoattractant for increasing WBCs to eliminate the infection [24]. However, leukocytosis cannot suppress MRSA colonization [25]. This was proven by the significant difference in a number of neutrophils compared with the lymphocytes. The increase in neutrophils was brought about by the CRP level and the immune expression of CD8+ and COX-2. These systemic changes are believed to be the impacts of MRSA because MRSA can be isolated from all the blood samples from the positive control. These mechanisms indicate that the systemic MRSA infection could worsen if not treated properly.

Vancomycin is the drug of choice for treating MRSA infections. In this study, MRSA was not isolated from the blood specimen of rats artificially infected with MRSA 7 days after vancomycin therapy. The rats treated using vancomycin still showed several impacts, including microcytic hypochromic anemia, thrombocytopenia, leukocytosis, neutrophilia, and increased CRP level; however, the decrease and/or increase in these parameters were not as high as in the positive control. In addition, the better prognosis after utilization of vancomycin to treat MRSA infection was indicated by the normalization of lymphocyte count, the percentage of immune expression of CD4+ and COX-2, and the decrease in the histopathological changes score of the observed tissues. Lymphocytes are critical for protection against the invasion of pathogenic infectious agents [26]. The normalization of lymphocyte counts and the balanced percentage of circulatory CD4+ indicated that the healing mechanism occurred. The decrease in the immune expression of COX-2, compared to the positive control, is also related to this. As an inflammatory biomarker, the normalization of COX-2 expression in the bloodstream indicated that systemic inflammation is subsiding [27].

The immune expression of CD8+ decreased compared to the positive control, which indicates that the infection was handled appropriately. This is also supported by the histopathology of the vancomycin group, which was not as severe as the positive control (p≤0.05). Vancomycin inhibits the biosynthesis of the cell membrane of MRSA by forming noncovalent hydrogen bonds [28]. The inhibition of MRSA cell membrane synthesis disrupts host-pathogen interaction. However, the utilization of vancomycin as a treatment against MRSA remains worrying as it poses the risk of this bacteria developing resistance. A previous study described that several clinical isolates can generate vancomycin-intermediate resistant *S. aureus* and vancomycin-resistant *S. aureus* [29].

Celery was chosen as the alternative therapy against MRSA systemic infection compared to vancomycin in this study. Celery exhibits several potential benefits against MRSA due to its biochemical content. A previous study described that celery contains alkaloids, flavonoids, phenolic compounds, saponins, and tannins [7]. Phenolic compounds are the highest biochemical content of celery extract. Celery has been shown to inhibit MRSA growth in solid and broth media [30]. Based on this study, celery extract can be a potential antibacterial agent *in vivo*, especially against systemic MRSA infections. This was proven by the result that showed that MRSA cannot be isolated from the blood of rat models 7 days after therapy with 4 mg celery extract per kg BW.

In contrast, MRSA can still be isolated after therapy using lower doses of celery extract. MRSA presence in the blood of the groups treated using lower doses of celery extract indicates that the growth of MRSA can be inhibited, but not as well as with the highest dose (4 mg celery extract per kg BW). Further analysis showed that celery extract, in all doses, repaired the L/N ratio and the immune expression of CD4+ and COX-2 in the circulatory system. Statistically, no significant differences were found regarding those parameters in all the groups treated with celery extract. In addition, treatment using celery in the highest doses caused some repairs, such as the normalization of MCHC, neutrophil count, and immune expression of COX-2, compared to the negative control. The potency of the celery extract was also determined through the inhibition of bacterial colonization in the infected hosts and through blood profile normalization.

The mechanism of celery in inhibiting the pathogenesis of MRSA infection is related to the antioxidant content of the celery extract. The biochemical compounds found in celery extracts, such as alkaloids and saponins, decrease the oxidative stress and redox-dependent pathway to prevent the severity of infection [31]. The decrease of oxidative stress triggers a more specific immune response to counter the infection [32]. Further, the activity of exogenous antioxidants derived from celery extract induces a balance in the endogenous antioxidants within the pericytes to maintain membrane integrity and hinder bacterial cell attachment. Failure of MRSA to attach to walls of blood vessels decreases the risks of infections [33]. Inhibiting MRSA and pericyte interaction prevents severe tissue changes histopathologically, which was shown by the better histopathological scores of the celery extract groups than the positive control. Next, histopathology changes were minimized with an increase in celery extract dose. In this study, the utilization of different doses of celery extract elucidated that systemic protection requires gradual processes in protection, healing, and repair.

## Conclusion

Celery alcoholic extract demonstrates the potential to be used as an antimicrobial agent against systemic MRSA infections. Celery extract showed protection from MRSA infection, especially from severe tissue destruction, and repaired the blood profile in infected rats. The utilization of celery extract cannot promote the direct elimination of systemic MRSA infections, but better results were observed with higher doses. A clinical study regarding the efficacy of celery extract must be conducted to ensure its potency against MRSA infections in humans.

## Authors’ Contributions

YAP and ADW: Study design, experimental procedure, and drafted and revised the manuscript. Both authors read and approved the final manuscript.
